# The diversity and abundance of chytrids on the Greenland Ice Sheet

**DOI:** 10.1038/s41598-026-41468-5

**Published:** 2026-02-26

**Authors:** Laura Perini, Athanasios Zervas, Louise Feld, Carsten S. Jacobsen, Liane G. Benning, Martyn Tranter, Alexandre M. Anesio

**Affiliations:** 1 Department of Environmental Science, Aarhus University, Roskilde, 4000 Denmark; 2 GFZ, Helmholtz Centre for Geosciences, 14473 Telegrafenberg, Potsdam, Germany; 3 Department of Earth Sciences, Freie Universität Berlin, 12249 Berlin, Germany

**Keywords:** Chytridiomycota, Glacier ice algae, Greenland Ice Sheet, Fungal diversity, Potential novel taxa, Chytrids radiation, Ecology, Ecology, Evolution, Microbiology

## Abstract

Zoosporic fungi of the Chytridiomycota phylum are lethal parasites of algae in Arctic freshwater and marine environments. The presence of members of the Chytridiomycota phylum has also been confirmed by amplicon sequencing and microscopy in various habitats on the Greenland Ice Sheet. Glacier ice algae of the genus *Ancylonema* (Zygnematophyceae, Streptophyta) dominate microbial communities on the margins of the Greenland Ice Sheet and play key roles in darkening and melting of surface ice. Recent studies and in-situ observations have identified undescribed chytrids infecting *Ancylonema* spp. blooms on the Greenland Ice Sheet^1^. In this study, the diversity and abundance of Chytridiomycota fungi were assessed by collection of ice surface and cryoconite holes samples from several locations on the ice sheet. We provide the first quantitative assessment, using qPCR, to reveal that uncultured chytridiomycetous fungi on dark ice were one order of magnitude higher than in cryoconites throughout the 2022 melt season on the Central-West margin of the Greenland Ice Sheet. Phylogenetic trees, based on the assembled environmental 18S rRNA genes, highlight that surface ice habitats dominated by glacier ice algae or snow algae harbor a great diversity of fungi belonging to the Blastocladiomycota, Monoblepharidomycota and Chytridiomycota phyla. They appear to be potential novel taxa, based on small subunit (18S) rRNA gene sequence similarity analysis, increasing our knowledge of chytrids diversity in Arctic environments. The number of novel lineages distinguished from described taxa varied from 63 to 81, based on two sequence identity thresholds (> 97% and > 98%), respectively. Overall, this study addresses the lack of quantitative and phylogenetically resolved information on chytrids in supraglacial environments by providing the first assessment of their abundance and diversity across the Greenland Ice Sheet.

**Supplementary Information:**

The online version contains supplementary material available at 10.1038/s41598-026-41468-5.

## Introduction

The Chytridiomycota phylum, commonly known as chytrids^1^, consists of a group of fungi whose members are ubiquitous in a range of soils, freshwater and marine ecosystems^2^. The Chytridiomycota *sensu lato* were recently divided into 6 phyla: Olpidiomycota^3^, Sanchytriomycota^4^, Monoblepharidomycota, Neocallimastigomycota, Blastocladiomycota, and Chytridiomycota *sensu stricto*^5–7^. The latter, in turn, is divided into several orders, including Polychytriales, Cladochytriales, Mesochytriales, Zygophlyctidales, Caulochytriales, Rhizophydiales, Gromochytriales, Lobulomycetales, Chytridiales, Synchytriales, Polyphagales, Spizellomycetales, Rhizophlyctidales, and Zygorhizidiales^7^. Chytrids are similar to other zoosporic fungi in that they produce flagellated zoospores and exhibit different trophic strategies (i.e., parasitism, saprotrophy)^8^.

**Fig. 1:**
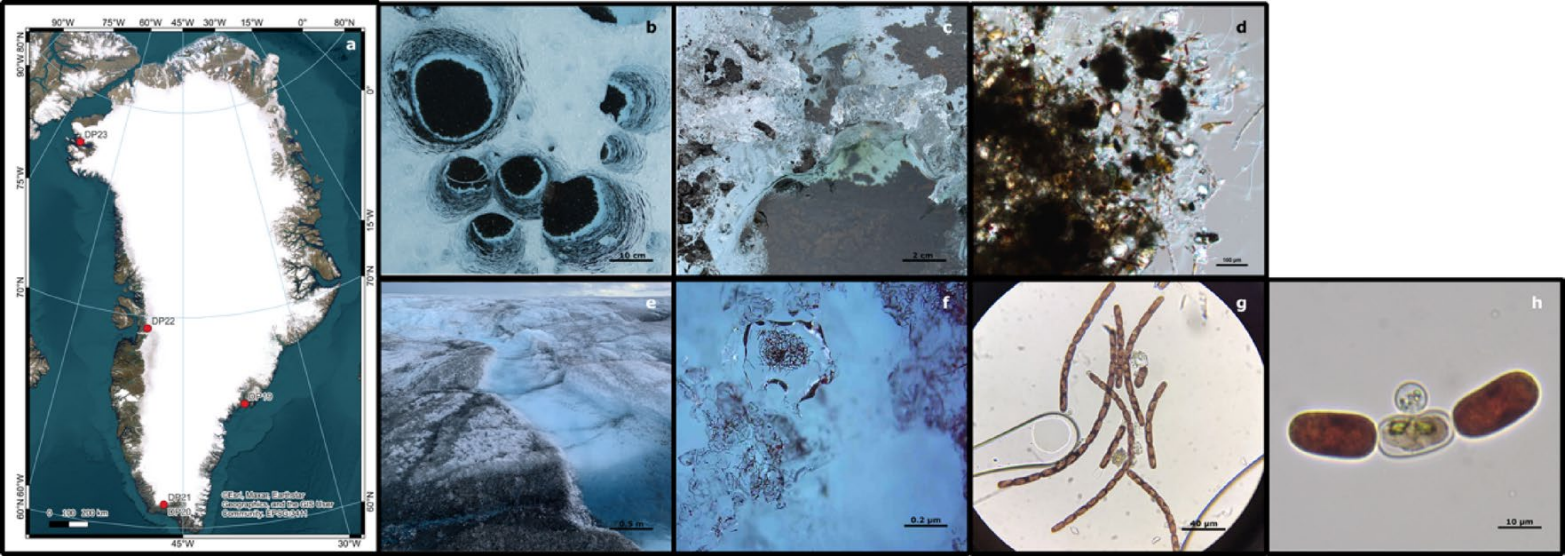
Increasingly smaller scales showing a satellite image with red dots marking locations on the GrIS where glacier ice algae blooms have been previously documented (**a**), cryoconite holes (**b**), sediment detail of a cryoconite hole (**c**) light microscopic image of cryoconite granules (**d**), dark ice surface ice (**e**), glacier ice algae on ice crystals, as recorded in the field with a handheld microscope (**f**), light microscopic image of *A. nordenskioeldii* (**g**),and a microalgae infected by an undescribed chytrid taken from the 2022 field campaign in Ilulissat, CW Greenland (**h**). Map layers were created using Esri, Maxar, Earthstar Geographics, and the GIS User Community by Dr Shunan Feng. EPSG:3411. https://www.qgis.org/downloads/windows/QGIS-OSGeo4W-3.30.0-1.0.0.0.msi.

Microbial life flourishes on glaciers and ice sheets, despite the extreme conditions, such as high light irradiation, low temperatures, and low nutrient availability. However, quantification and diversity assessment of chytrids on supraglacial ice surfaces are still lacking. Despite evidence from culture independent surveys that chytrids occur in cold habitats, including the supraglacial weathering crust, snow fields, cryoconite hole sediments, glacial freshwater runoff or in periglacial soils from glaciers and snowpacks worldwide^9–14^, comprehensive assessments of their diversity in supraglacial ice environments remain scarce^15^^[,16^,

Microbial communities on supraglacial systems are diverse and spread across a variety of habitats, including the weathering crust (i.e., top centimetres of surface ice, known as the living skin of glaciers), snow cover, and cryoconite holes (i.e., water-filled depressions containing a basal mineral and organic sediment^17^ that serve as hot-spots of microbial activity and reduce ice albedo, thereby enhancing melt). The weathering crust during summer, in the ablation area of the Greenland Ice Sheet (GrIS), is inhabited predominately by the dark pigmented glacier ice algae *Ancylonema nordenskioeldii* and *A. alaskanum*^18–20^ (Fig. 1). While recent extensive efforts have increased knowledge on the physiology, ecology, and phylogeny of these primary producers, relatively little is known about the broader microbial interactions and community structures that co-occur with glacier ice algae^21–24^. Generally, viruses, grazers and parasitic fungi act as growth controllers in algal blooms in aquatic environments^8,25–27^. Recent studies conducted on a variety of habitats in the GrIS highlighted that the giant viruses which infect microeukaryotes might be hosted by snow algae rather than glacier ice algae^1^. Furthermore, grazers, such as tardigrades and rotifers, are commonly found in cryoconite hole sediments, but less so in the weathering crust on the GrIS^28^. This distribution suggests that grazing pressure on glacier ice algae may be limited in surface ice habitats. Together, these observations raise the possibility that neither giant viruses nor grazers are major regulators of glacier ice algal biomass.

Parasitic chytrids infecting *Ancylonema* spp. blooms on Alaskan glacier surface ice were first reported by Kol^29^. Studies conducted on other glaciers in Svalbard and Alaska corroborated these observations of parasitic fungi infecting *Ancylonema* spp. blooms^30,31^. Fiołka^30^, performed light and fluorescence microscopy on glacier ice algal cells and their parasitic fungi from Svalbard samples to identify the infecting fungal species through their morphological characteristics. Meanwhile, Kobayashi et al.^31^, focused on quantifying the prevalence of infection in algal blooms thriving on the weathering crust ice and in cryoconite holes on Alaskan glaciers and found that 4% of glacier ice algae on the ice surface and 20% in cryoconite holes were infected by chytrids. Recently, we demonstrated through laboratory experiments the presence of parasitic fungi belonging to the Chytridiomycota phylum infecting *Ancylonema* spp. algae from Greenland^14^.

In this study we characterize the spatiotemporal patterns of chytrid abundance and community composition in supraglacial ice across four summers in Greenland. We used TotalRNA and metagenomic data to assemble full-length 18S rRNA gene sequences from weathering crust, snow, and cryoconite hole habitats and document a wide diversity of chytrids, with many potential novel taxa. We do not directly quantify infections or host–parasite associations; therefore, we interpret our results at the community level and view potential impacts on algal blooms as hypotheses for future work. Our dataset therefore provides baseline information on the diversity and distribution of chytrids and their potential ecological significance in glacial ecosystems.

## Materials and methods

### Sample collection

**Fig. 2:**
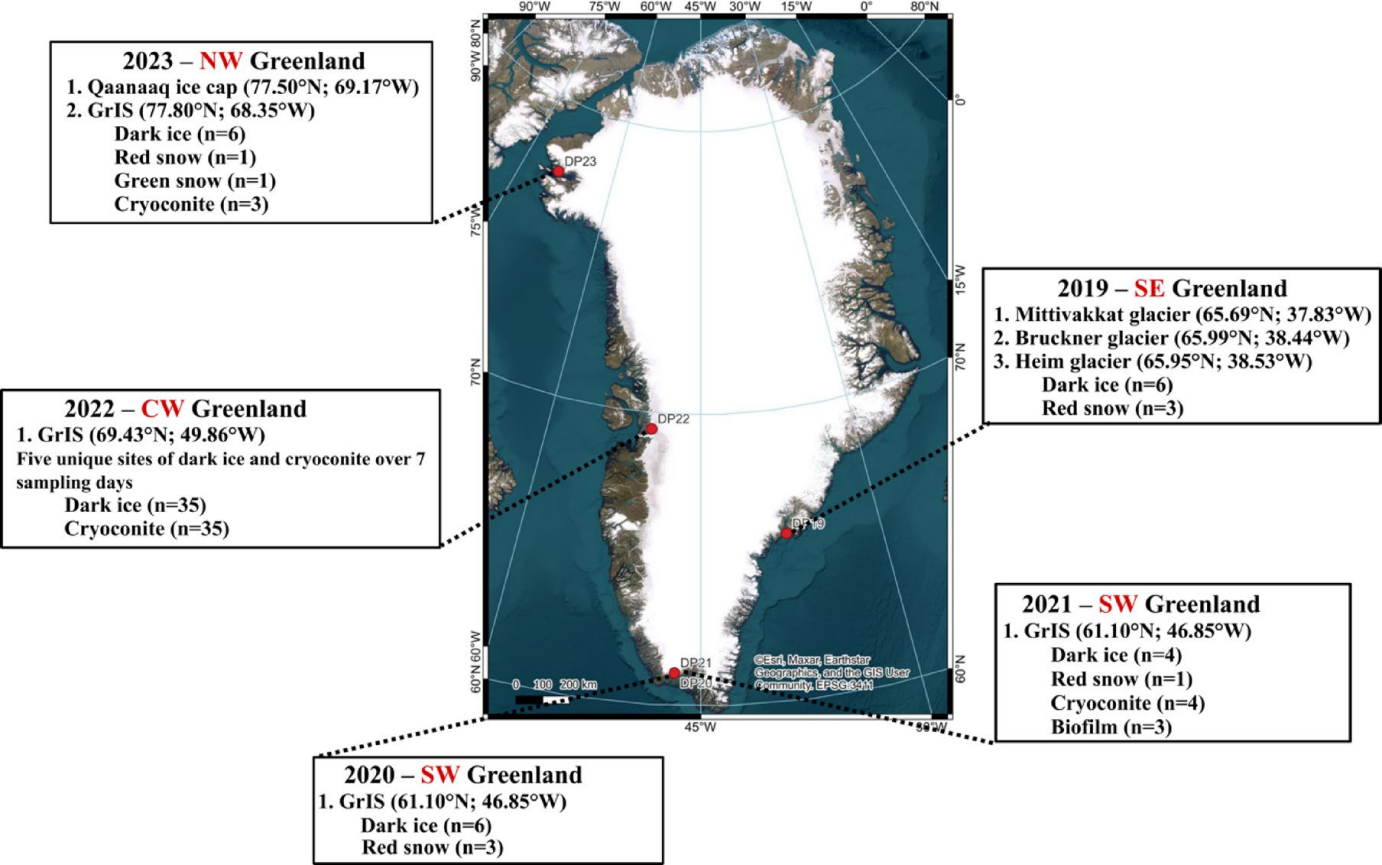
Locations of fieldwork campaigns from 2019 to 2023 melting seasons on the Greenland Ice Sheet, with details on the glaciers and habitats sampled. NW stands for North-West, CW stands for Central-West, SW stands for South-West, and SE stands for South-East. Map layers were created using Esri, Maxar, Earthstar Geographics, and the GIS User Community by Dr Shunan Feng. EPSG:3411. https://www.qgis.org/downloads/windows/QGIS-OSGeo4W-3.30.0-1.0.0.0.msi DP stands for Deep Purple (https://www.deeppurple-ercsyg.eu/).

Samples were collected during four fieldwork campaigns between July and August, from 2019 to 2023 (Fig. 2).

In 2019, samples were collected at three locations in SE Greenland, near Tasilaq: Mittivakkat (65.69°N; 37.83°W), Bruckner (65.99°N; 38.44°W), and Heim glaciers (65.95°N; 38.53°W) (Table S1). These samples are also described in Halbach et al.^32^, and in Perini et al.^1^. Samples included weathering crust ice, colored visibly by the high abundance (10^4^ cell/ml) of dark pigmented microalgae of the class Zygnematophyceae (dark weathering crust ice; *n* = 6), and snow samples dominated by red pigmented microalgae of the class Chlorophyceae (red snow; *n* = 3).

In 2020 and 2021, samples were collected near Qaqortoq in SW Greenland, close to the QAS_U and QAS_M PROMICE Stations (61.10°N; 46.85°W), as previously described in Jaarsma et al.^33^, and Perini et al.^1 ^,(Tables S1 and S2). The samples collected in the 2020 season consisted of cryoconite sediment (*n* = 1), dark weathering crust ice (*n* = 1), green snow (*n* = 2), and red snow (*n* = 2). In the 2021 season, samples included dark weathering crust ice (*n* = 4), red snow (*n* = 1), biofilm (*n* = 3), and cryoconite hole sediment (*n* = 4).

In 2022, samples were collected in Central West Greenland, Northeast of Ilulissat (69.43°N; 49.86°W) (Table S3). The samples comprised of dark weathering crust ice from five different sites and five cryoconite hole sediments, sampled on seven different occasions (over 21-days), giving a total of 35 samples for each habitat. (*n* = 35 ice, 35 cryoconite hole sediment). Sampling days were 28.07.2022 (day 209 of the Julian calendar), 01.08.2022 (day 213), 05.08.2022 (day 217), 07.08.2022 (day 219), 10.08.2022 (day 222), 13.08.2022 (day 225), 18.08.2022 (day 230).

In 2023, samples were collected in NW Greenland, close to Qaanaaq (77.52°N; 69.07°W) (Table S4). The samples included material from the Qaanaaq ice cap (77.50°N; 69.17°W) and the Greenland ice sheet (77.80°N; 68.35°W). Dark weathering crust ice was sampled on both the Qaanaaq ice cap (*n* = 3) and the Greenland ice sheet (*n* = 3). Red and green snow (*n* = 1 of each), and cryoconite hole sediment (*n* = 3) were only collected on the Qaanaaq ice cap. All samples representing dark weathering crust ice are hereafter called “dark ice”, all samples representing cryoconite hole sediment material will be hereafter called ‘cryoconite’ and samples representing pigmented snow will either be called ‘red snow’ or ‘green snow’.

Coordinates and full details for each sampling site and each sample are detailed in the supplementary material (Table S1-S4). All the samples were collected using sterile nitrile gloves and tools and subsequently stored in sterile Whirl-Pak bags.

### Sample preparation and sequencing

Sample preparations from the different field campaigns followed a consistent protocol, as described below. Extensive details on the extraction of nucleic acids, library preparation and sequencing are provided in^32^ and Perini et al.^1^, for the 2019–2020 dataset, and in Jaarsma et al.^33^, for the 2021 dataset. Sample processing for the 2022 and 2023 datasets, followed the same protocols as in Jaarsma et al.^33^. Briefly, cryoconite hole material was collected with a large polycarbonate pipette, transferred into sterile 4 mL cryotubes and flash frozen in a liquid nitrogen cooled dry shipper. Ice samples were collected by scrapping approximately 2 L of the upper 2–5 cm of the weathering crust ice with a field-sterilized ice axe into sterile Whirl-Pak bags that were melted at 10 °C^34^. Approximately 600 mL of the melted ice was filtered through single use sterile cellulose nitrate filters (0.2 μm). The biomass laden filters were subsequently rolled up, placed in a cryotube and stored in the −196 °C cryoshipper. For snow samples the top few millimeters of the coloured snow were collected and all other sample handling procedures were the same. Because they contained a high amount of material, the filters in the cryotubes containing cryoconite hole sediment were freeze-dried prior to further use. RNA/DNA was co-extracted from 200 mg of freeze-dried cryoconite hole sediment material or half filters of ice/snow (cut with sterile micro-dissecting scissors in the lab) using the Nucleobond RNA Soil Mini kit and the accompanying DNA co-extraction module (Macherey-Nagel, Germany) following the manufacturer’s instructions. RNA/DNA concentrations were measured on a Qubit 4 (Thermo Fisher Scientific) using the Qubit RNA HS Assay Kit and the Qubit 1X dsDNA HS Assay Kit (Thermo Fisher Scientific), respectively.

Metagenomic sequencing libraries were generated with the NEBNext Ultra II FS DNA Library Prep Kit (Illumina), with 8 rounds of PCR amplification following the manufacturer’s instructions (New England Biolabs, USA). RNA samples were treated with the RapidOUT Kit to eliminate any remaining DNA (Thermo Scientific), according to manufacturer’s instructions. RNA libraries were prepared using the NEBNext Ultra II RNA library Kit, with 8 rounds of PCR amplification following the manufacturer’s instructions (New England Biolabs, USA). Metagenomic and TotalRNA sequencing was performed on either MiSeq or NextSeq Illumina platforms in 150 pair-end mode. Assembly type is specified for each sample of each sampling in Tables S1-S4.

Raw Illumina reads were processed using our in-house TotalRNA workflow (version 1.1.0 (DOI:10.5281/zenodo.7656004) as described elsewhere^33^^[,35^. The reconstructed, full-length rRNA small subunit (18S) genes in the metagenomic and metatranscriptomic data were taxonomically identified with Silva 138.1 and sequences assigned to phylum Chytridiomycota, Blastocladiomycota, and Monoblepharidomycota were extracted for subsequent analyses.

In the 2022 season (CW Greenland, Northeast of Ilulissat), samples from the same weathering crust patches and cryoconite hole sediments were collected over 21-days. Therefore, relative abundances of the active Chytridiomycota from 2022 season were used to build line graphs showing the temporal and spatial variation in abundance of the Chytridiomycota community. Analyses were performed using R studio v2023.12.0 + 369 and R v4.3.2 (R Core Team (R Foundation for Statistical Computing)^36^.

### Phylogeny of environmental rRNA 18S genes

**Fig. 3:**
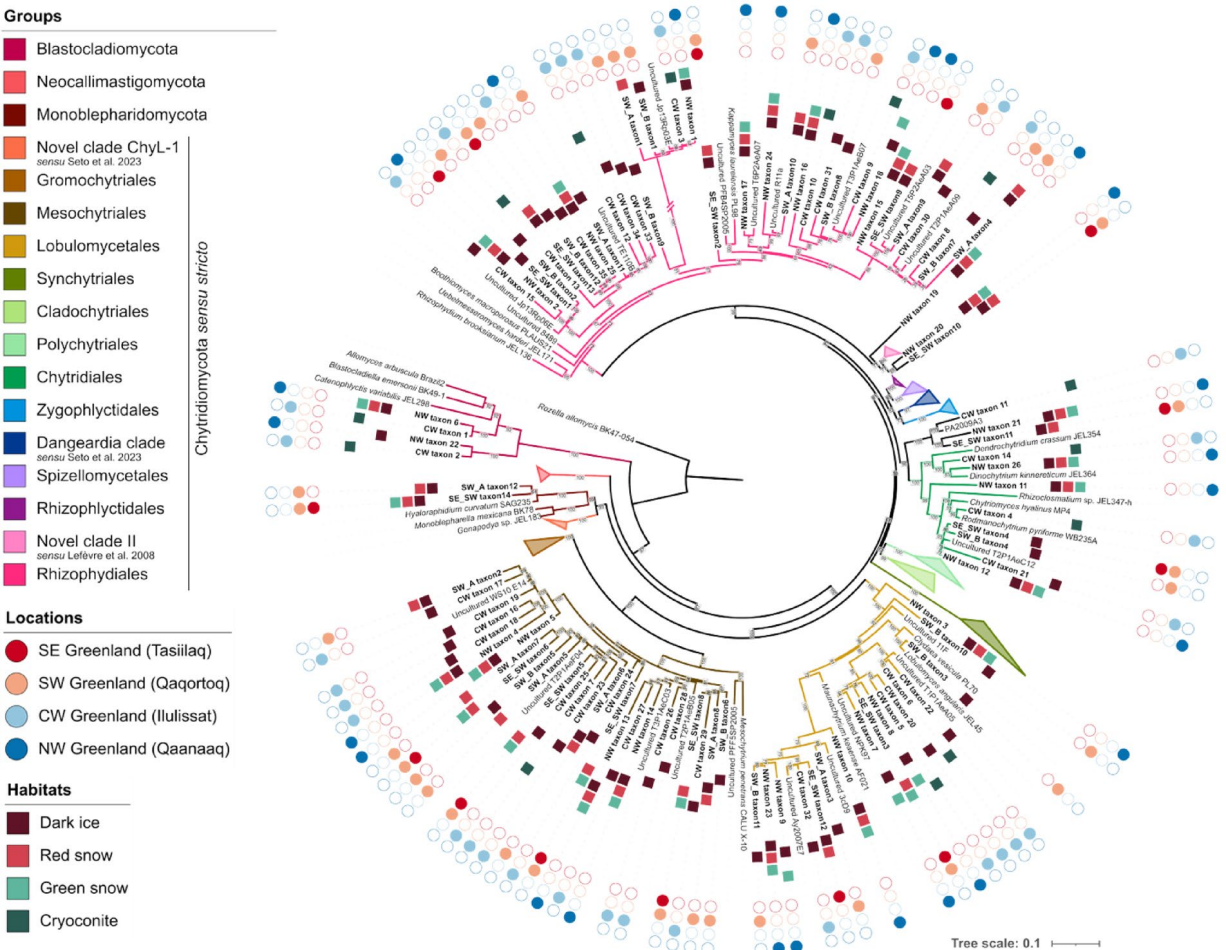
Maximum likelihood tree showing the phylogenetic position of chytrid species belonging to Blastocladiomycota, Monoblepharidomycota and Chytridiomycota phyla using a concatenated alignment of the 18S–5.8S-28S rRNA genes. ML bootstrap values higher than 50% were shown on each branch. Sequences recovered from the environmental samples are presented in bold. Groups that did not include sequences from our study were collapsed. Double slashes on branches indicate that length is reduced by half. Squares of each taxa indicate the habitats from which they were sequenced. Outer ring indicates the locations from which each sequence was retrieved.

Full-length rRNA 18S genes from all samples identified as Chytridiomycota, Blastocladiomycota, and Monoblepharidomycota phyla were aligned against 77 concatenated reference sequences of the 18S, 5.8S and 28S rRNA genes (Table S5) to clarify their phylogenetic position. In the concatenated dataset, the order of the rRNA gene sequences was the following: 18S, 5.8S and 28S, and the sequences were manually separated by 5 gaps. The alignment was performed using MAFFT^37^ v7.475 using the following options: --auto and --maxiterate 1000. Pairwise distance similarity matrices were calculated in Geneious (Geneious Prime 2024.0.7 (https://www.geneious.com). The maximum likelihood (ML) tree was built using IQ-TREE^38^ v2.0.3. According to BIC scores, GTR+F+I+G4 was the best model by the “-m TEST” ModelFinder option^39^. To assess confidence, IQ-TREE was run with 1000 ultrafast bootstraps (-alrt 1000 -B 1000)^40^. To ensure a consistent tree topology, we generated an additional ML tree by combining our environmental full-length 18S rRNA genes with a recently published dataset of 18S rRNA sequences from 237 reference species and uncultured taxa^7^. Alignment and tree were performed as described above (best model according to BIC score: GTR+F+I+G4) and can be found in the supplementary material (Figure S1). Since the topology of the two ML trees did not change, we decided to show the easier to read (Fig. 3). The other is shown in supplemental information (Figure S1).

### Quantitative assessment of uncultured chytrids by qPCR

The temporal and spatial abundance of chytrids was assessed from extracted DNA of both dark ice and cryoconite sediment of the 2022 fieldwork campaign. The DNA was amplified with the F-Chyt (5’- GCAGGCTTACGCTTGAATAC − 3’) and R-Chyt (5’- CATAAGGTGCCGAACAAGTC − 3’) primer set ^41^, targeting a region in the 18S rDNA specific to chytrids. Primer coverage was assessed in silico using the ‘Test with Saved Primers’ function in Geneious (Geneious Prime 2024.0.7 (https://www.geneious.com). Primers were evaluated against the set of sequences used to construct the phylogenetic tree shown in Fig. 3 of the manuscript. The specificity of the primers in our samples was tested using a range of 8 non-targeted organisms (Table S6). Although weak amplification of some non-target organisms was observed, it occurred only at very high C_q_ values (C_q_ 36–40), whereas all ice and cryoconite samples amplified much earlier (C_q_ 17–27), indicating negligible contribution of non-target DNA. DNA extraction was unsuccessful for 4 cryoconite sediment samples (cc1 day3, cc1 day5, and cc5 day5) out of 35, and these were excluded from this analysis.

The qPCR reaction mix contained 10 µl of 2× qPCRBIO SyGreen Blue Mix (PCR Biosystems), 0.4 µM of each forward (F-Chyt) and reverse primers (R-Chyt) and 2 µl DNA sample in a final reaction volume of 20 µl. The concentration of DNA was an order of magnitude higher in samples extracted from cryoconite sediment (10–438 ng/µl) than from ice (0.1–39 ng/µl). Thus, all samples from sediment were diluted 10-fold in milliQ water before being used as template in the qPCR reactions to obtain more comparable DNA template concentrations.

The qPCR reactions were carried out in a BioRad CFX Connect Real-time system using the cycling conditions; 95 °C for 2 min, 40 cycles of 95 °C for 5 s and 66 °C for 30 s, followed by a melt curve analysis from 66 to 95 °C with 0.1 °C increment steps.

A standard was prepared from *Boothiomyces macroporosum* CBS 122107 obtained from The Westerdijk Fungal Biodiversity Institute (NL) for quantification of the chytrid copy number. In short, DNA was extracted from the strain culture using the Fast DNA Spin Kit (MP Biomedicals) according to manufacturer instructions. DNA from the standard strain was then used as template to set up five individual PCR reactions using the same mastermix and cycling conditions as described above with the addition of a final extension at 72 °C for 30 s and omitting the melt curve analysis. The PCR products from the five reactions were pooled and cleaned with NEBNext beads according to manufacturer instructions. The size of the PCR product was verified using Tapestation D5000 and the concentration determined using the Qubit High Sensitivity DNA kit. The purified PCR product representing a 309 bp region of the *B. macroporosum* 18S rDNA sequence was used to prepare 10-fold dilutions (5 to 5000000 copies/µl), which were then used as qPCR standard in technical triplicates. The quantifications of Chytridiomycota in all dark ice and cryoconite sediment samples were based on triplicate qPCR reactions.

The relative abundances of the Chytridiomycota were calculated against the overall microbial community from the 2022 summer campaign using R (R Core Team (R Foundation for Statistical Computing)^36) ^(Table S7). qPCR analyses were normalized to a single-copy standard, so the results reflect total 18S rRNA gene copy numbers rather than abundance of specific taxa. We assume that chytrids as a group have a similar average copy number in the absence of taxon-specific data. We note that more precise estimates for individual taxa will require further studies on copy number variation across chytrid lineages.

## Results

### Phylogenetic position of 18S rRNA environmental genes

The metagenomic and metatranscriptomic analysis of samples collected from the various supraglacial habitats, namely red snow, green snow, dark ice, and cryoconite sediment, across four locations in Greenland (SE, SW, CW and NW) generated 99 full-length 18S rRNA gene sequences identified as belonging to Blastocladiomycota, and Chytridiomycota phyla, according to classification with Silva v138.1.

The ML tree of the 18S–5.8S-28S rDNA concatenated dataset assigned the environmental sequences within the Chytridiomycota phylum to four orders: Rhizophydiales (35.4% of the total sequences), Mesochytriales (27.3%), Lobulomycetales (17.2%), and Chytridiales (8.1%) (Fig. 3, Table S8). A few sequences were also identified as belonging to the Monoblepharidomycota (2%) and Blastocladiomycota (4%) phyla, and to *incertae sedis* (6%) (Table S8). In few cases, the environmental sequences were closely related, despite originating from different locations and habitats (Fig. 3). For example, a high percentage of identity was found between sequences retrieved from SE, SW, CW and NW Greenland in the Rhizophydiales (97.9*–*98.6% of identity), in the Chytridiales (98*–*98.9%), and in the Lobulomycetales (97.1*–*99.4%) orders. Pairwise distance matrices can be found in Table S9-S14.

Most of the taxa were assigned to the Rhizophydiales (28.6*–*45.8%), followed by the Mesochytriales (15.4*–*34.3%), in all the locations, except for NW Greenland, where the second most present order was the Lobulomycetales (23.1%) (Table S8).

**Table 1:** Numbers of taxa present in surface ice habitats (dark ice, red or green snow) and cryoconite sediment based on a 98% identity cut-off.

Taxa	Presence in red/green snow and dark ice	Presence in cryoconite	Presence in both habitats
Phylum – Blastocladiomycota	1	1	1
Phylum – Monoblepharidomycota	2	0	0
Order – Rhizophydiales	19	5	1
Order – Mesochytriales	24	1	0
Order – Chytridiales	6	3	0
Order – Lobulomycetales	13	2	0
Incertae sedis	5	1	0

The majority of taxa were present in both surface habitats (red/green snow and dark ice) (84.3%) and less so in cryoconite (15.7%), with only 2 taxa shared between the two habitats (Table 1).

### Taxa assigned to known species and uncultured strains (98% cut-off)

Only 2 out of a total of 99 sequences were identified as previously described species. Sequence SW_B taxon3, retrieved from dark ice in SW Greenland was classified as *Lobulomyces angularis* (99.2% of identity). The only known strain, *Lobulomyces angularis* JEL45, was isolated from an acidic freshwater lake in Maine (USA) and exhibits a saprotrophic lifestyle^42^. Sequence CW taxon4 from cryoconite sediments from CW Greenland was classified as *Rodmanochytrium pyriforme* (98.6% of identity). The only known strain, *Rodmanochytrium pyriforme* WB235A, was isolated from a freshwater lake in Alabama, USA, and also has a saprotrophic lifestyle^43^.

Several environmental sequences retrieved from dark ice, green and red snow, and cryoconite sediments shared a high identity (with a bootstrap support ranging from 98 to 100%) with other uncultured strains sequenced from freshwater lakes in Japan and France^44,45^, or from other cold habitats, including periglacial soil (Himalaya, Nepal, and Rocky Mountains, USA), glacial ice (Svalbard) and sea ice (Sweden)^10,46,47^(Table 2; Fig. 3).

**Table 2:** List of taxa identified as known species or showing a high percentage of identity with environmental uncultured strains ordered by family, taxa and location based on the concatenated alignment of the 18S–5.8S-28S rRNA genes (Fig. 3). In parentheses in the order column there is specified the ratio between taxa identified and total number of taxa assigned to the specific order. 98% of identity was used as threshold. SEG stands for South-East Greenland; SE-SWG stands for South-East and South-West Greenland; SWG stands for South-West Greenland; CWG stands for Central-West Greenland; NWG stands for North-West Greenland. DI stands for dark ice; GS stands for green snow; RS stands for red snow; CC stands for cryoconite.

Order	Taxa	Location	Habitat	Identity %	GenBank accession no. (18S rDNA)	Known species/Uncultured strain	Habitat/Geographic location	Characterization	References
Mesochytriales (1/27)	*CW taxon28*	CWG	DI	98.5	GQ995413	T3P1AeC03	Periglacial soil, Nepal and USA	-	Freeman^10^,
Rhizophydiales (11/35)	*SE taxon1*	SEG	DI	98.5	AB971114	Jp13Rp06E	Freshwater lake, Japan	-	Ishida et al.^44^,
	*SE taxon9*	SEG	DI, RS	98.4	GC995423	T5P2AeA03	Periglacial soil, Nepal and USA	-	Freeman^10^,
	*SW_A taxon1*	SWG	RS	98	AB971109	Jp13Rp03E	Freshwater lake, Japan	-	Ishida et al.^44^,
	*SW_A taxon9*	SWG	DI, RS	98.5	GC995423	T5P2AeA03	Periglacial soil, Nepal and USA	-	Freeman^10^,
	*SW_B taxon1*	SWG	DI	99.2	AB971109	Jp13Rp03E	Freshwater lake, Japan	-	Ishida et al.^44^,
	*SW_B taxon2*	SWG	DI	98.2	AB971114	Jp13Rp06E	Freshwater lake, Japan	-	Ishida et al.^44^,
	*CW taxon3*	CWG	CC	99.7	AB971109	Jp13Rp03E	Freshwater lake, Japan	-	Ishida et al.^44^,
	*CW taxon30*	CWG	DI	98	GQ995427	T2P1AeA09	Periglacial soil, Nepal and USA	-	Freeman^10^,
	*NW taxon1*	NWG	DI, GS	99.6	AB971109	Jp13Rp03E	Freshwater lake, Japan	-	Ishida et al.^44^,
	*NW taxon15*	NWG	DI, RS, GS	98.2	GQ995427	T2P1AeA09	Periglacial soil, Nepal and USA	-	Freeman^10^,
	*NW taxon17*	NWG	DI, RS, GS	98.6	GQ995432	T6P2AeA07	Periglacial soil, Nepal and USA	-	Freeman^10^,
Chytridiales (4/8)	*SE-SW taxon4*	SE-SWG	DI	98.7	GC995264	T2P1AeC12	Periglacial soil, Nepal and USA	-	Freeman^10^,
	*SW_B taxon4*	SWG	DI	98	GC995264	T2P1AeC12	Periglacial soil, Nepal and USA	-	Freeman^10^,
	*CW taxon 4*	CWG	CC	98.6	DQ536486	*Rodmanochytrium pyriforme* WB235A	Freshwater lake, USA	Saprophytic lifestyle	Powell^43^,
	*NW taxon12*	NWG	DI, RS, GS	98.9	GC995264	T2P1AeC12	Periglacial soil, Nepal and USA	-	Freeman^10^,
Lobulomycetales (6/17)	*SE taxon12*	SEG	DI, RS	98.3	JQ689413	Ay2007E7	Eutrophic lake Aydat, France	-	Jobard^45^,
	*SE-SW taxon3*	SE-SWG	RS, GS	98.1	EU371354	NPK97-82	Glacial ice, Svalbard	-	Luo et al.^46^,
	*SW_A taxon3*	SWG	DI	98.2	FN690491	3cD9	Sea ice, Sweden	Likely parasitic lifestyle	Majaneva^47^,
	*SW_B taxon3*	SWG	DI	99.2	AF164253	*Lobulomyces angularis* JEL45	Acidic freshwater lake, USA	Saprophytic lifestyle	Simmons et al.^42^,
	*CW taxon6*	CWG	CC	99.1	GQ995410	T1P1AeA05	Periglacial soil, Nepal and USA	-	Freeman^10^,
	*CW taxon32*	CWG	DI	98.6	JQ689413	Ay2007E7	Eutrophic lake Aydat, France	-	Jobard^45^,

### Determining potentially novel taxa (98% cut-off)

Numbers of potentially novel taxa were calculated based on the 18S gene percentage of similarity between taxa using two different standard cut-offs applied in different sequencing pipelines (97 and 98%). The first is a broad similarity cut-off based on the general approach used in the classification of OTUs (Operational Taxonomic Units) and is employed as a standard threshold for determining whether bacterial or other microorganism sequences belong to the same species. The second is based on our TotalRNA pipeline (specifically at the mapped to reads step) and introduces a more stringent option, allowing for a finer resolution in sequence classification.

In total, 81 novel 18S rRNA gene sequences (thereafter potentially undescribed taxa) were retrieved using a 98% similarity threshold, highlighting a high diversity of chytrids. We counted 25 different potentially undescribed taxa from the Rhizophydiales order. Some clusters assigned to this order contained sequences from samples collected in different locations and were > 99% similar with a branch support of 95.7% (e.g. SE-SW taxon13, SW_B taxon12, CW taxon35 and NW taxon25) or > 98% similar with a branch support of 100% (e.g. SW_A taxon1, SW_B taxon1, CW taxon3, NW taxon1).

In total, 25 potentially undescribed taxa of Mesochytriales were found, most of them (*n* = 11) were recovered from dark ice from CW Greenland (2022). Only three sequences from SE-SW and SW Greenland shared an identity value > 98% and were considered as the same species (SE-SW taxon8, SW_A taxon8, and SW_B taxon6) with a bootstrap support of 87%.

We found 8 undescribed taxa assigned to the Chytridiales. Taxa recovered from different locations of the GrIS (e.g. SE-SW taxon4, NW taxon12, SW_B taxon14) shared a high percentage of identity (> 98%, with a bootstrap support of 93–100%). The Lobulomycetales order contained 14 potentially undescribed taxa. As for the other orders, taxa assigned to the Lobulomycetales that were retrieved from different locations had a high identity (e.g. CW taxon32 and SE-SW taxon 12, 98.7% identity and 99% of bootstrap support, and SW_B taxon11 and NW taxon23, 99.4% identity and 80% of bootstrap support).

Two taxa assigned to the Monoblepharidomycota phylum were found in samples from SE-SW and SW Greenland locations. These two taxa (SE-SW taxon14 and SW_A taxon12) were 96.8 and 97.7%, respectively, similar to *Hyaloraphidium curvatum* SAG235. Therefore, they most likely represent two potentially novel undescribed taxa.

Only 4 taxa, which accounted for 3 different potentially novel ones (NW taxon6 and CW taxon1 had 98.1% identity), were assigned to the Blastocladiomycota phylum, 2 of which were retrieved from cryoconite sediments.

Three taxa were of *incertae sedis *and clustered closely to the “Novel Clade II”, as defined by Lefèvre et al.^48^.

The genetic distance of the 18S rRNA genes assigned to the Mesochytriales order showed that sequences retrieved from different locations of the GrIS (SW_B taxon6, SE-SW taxon8, CW taxon28, SW_A taxon6) and retrieved from dark ice, green and red snow habitats had a high percentage of bases (98–98.7%) identical to a novel group of snow algae infecting chytrids isolated from an alpine snow ecosystem in Japan^49^ (Figure S2). The pairwise genetic distance of all the environmental 18S rRNA genes, calculated against the only 18S sequences available of the parasitic species morphologically identified by Kol in the Alaskan glacier^29^, *Urceomyces sphaerocarpus* isolate ARG129, showed low identity (78.7–93.3%).

**Fig. 4:**
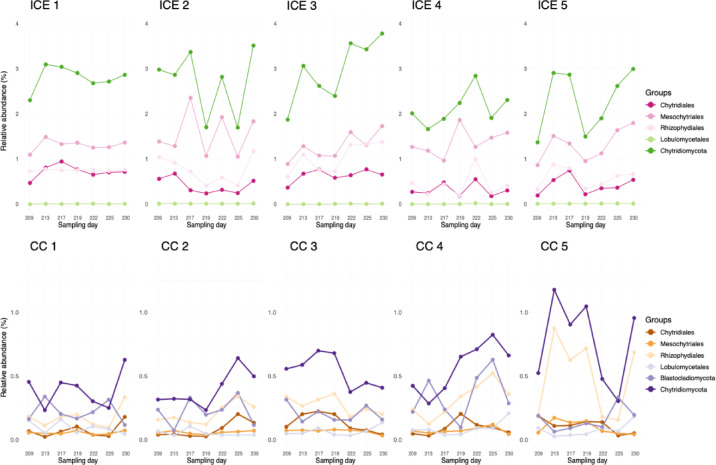
Dynamics of Chytridiomycota phylum (combination of all orders) and single orders over the 2022 melting season. Chytrids were quantified as relative abundance calculated against the whole microbial community for five samples of dark ice and cryoconite in 7 sampling days over a 21-day period. ICE stands for dark ice and CC stands for cryoconite.

### Known and unknown species with a 97% cut-off

We counted 63 potentially novel undescribed taxa with a cut-off of 97%. However, in this case, two species in the Lobulomycetales family, *Clydaea vesicula* and *Lobulomyces angularis*, cluster as a single species (identity 97.6%), together with taxon SW_B taxon3 (from SW GrIS 2022). Moreover, in the Monoblepharidomycota phylum, one taxon was identified as *Hyaloraphidium curvatum* SAG235 (97.7% of identity). In the Rhizophydiales, Chytridiales, Lobulomycetales and Mesochytriales orders, clusters of more than 3 sequences were identified as single taxa, reducing the number of potentially novel taxa from 81 (calculated with the 98% identity threshold) to 63.

### Chytrid dynamics during the 2022 melting season

**Fig. 5:**
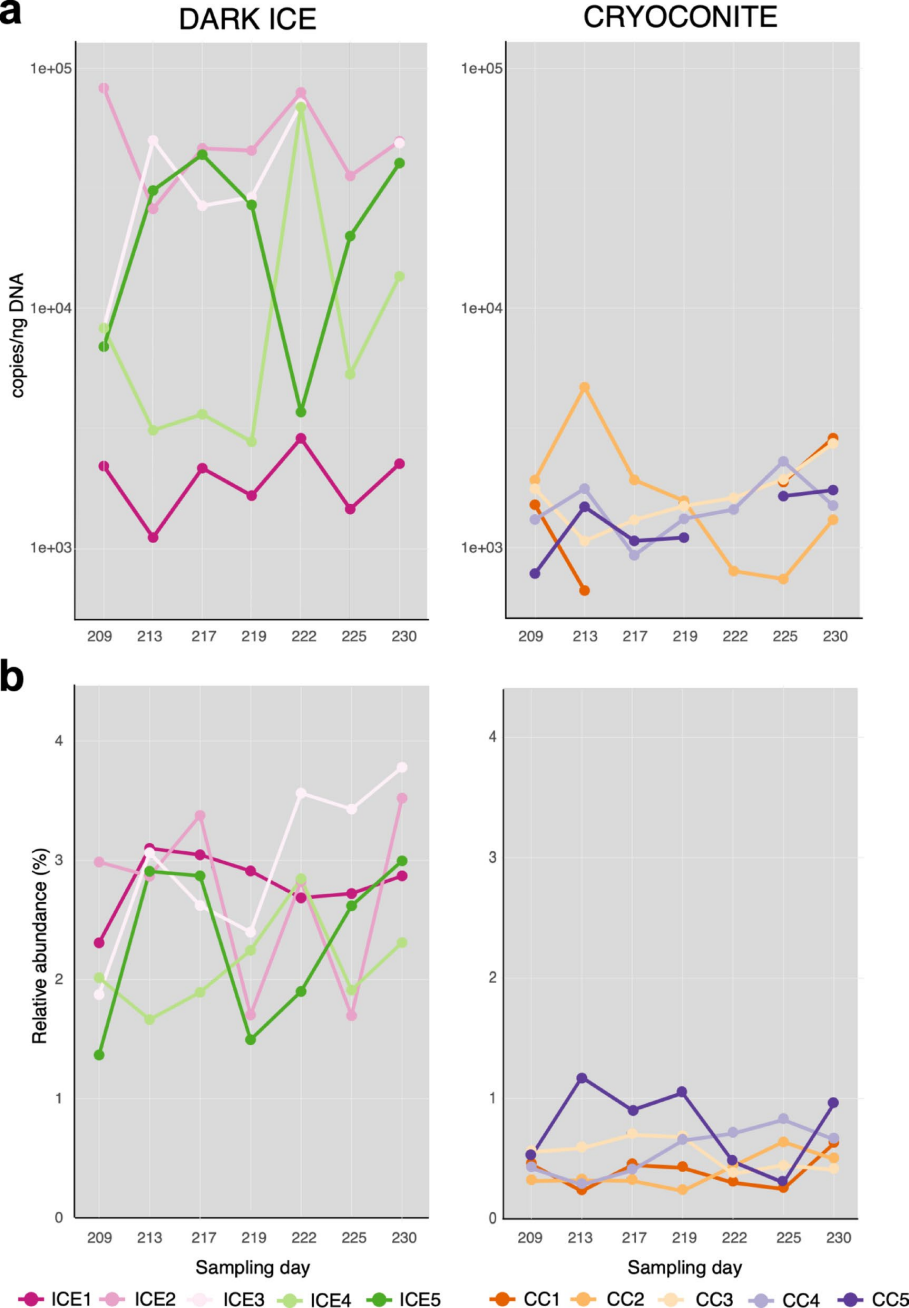
Dynamics of chytrids over the 2022 melting season. Chytrids were quantified as number of copies/ng DNA (**a**) and relative abundance calculated against the whole microbial community (**b**) for five samples of dark ice (left) and cryoconite (right) over 21-days.

Chytrids were quantified by qPCR using the number of copies/ng of extracted DNA in 5 dark ice and 5 cryoconite samples collected during 7 sampling events in a period of 21 days. Dark ice samples had a larger temporal variation and chytrid abundance (around one order of magnitude higher, 1.1 × 10^3^–1.4 × 10^5^) compared to cryoconite samples (6.6 × 10^2^–4.7 × 10^3^) (Fig. 5a, Table S15).

The average relative abundance of active Chytridiomycota in dark ice and cryoconite calculated against the whole microbial community was 2.6% and 0.5%, respectively. Temporal and spatial patterns in TotalRNA data were similar to the trends found using qPCR (Fig. 5b). Spatial relative abundances of the specific orders were similar in all dark ice samples, with Mesochytriales being the most abundant (average ranging between 1.28 and 1.56%), followed by Rhizophydiales (0.43–1.03%), Chytridiales (0.32–0.73%), and Lobulomycetales (0–0.01%). Spatial relative abundances of the specific orders were similar in the cryoconite samples, with Rhizophydiales and Blastocladiomycota being alternately the most abundant (average ranging between 0.17 and 0.49% and 0.16–0.34%, respectively), followed by Chytridiales (0.07–0.13%), Lobulomycetales (0.05–0.09%), and Mesochytriales (0.05–0.09%) (Fig. 4).

## Discussion

### Surface ice environments of the Greenland Ice Sheet harbor an undiscovered diversity of chytrids

Through this study we have assessed the diversity of chytrids in supraglacial, algae-dominated and cryoconite habitats, highlighting potential parasitic fungal species that could act as top-down controls of glacier ice algal and snow algal blooms that darken the surface and enhance melting. Analyses of the full length 18S rRNA metagenomic and metatranscriptomic data from surface habitats from different locations of the Greenland Ice Sheet revealed the presence of a considerable previously undescribed diversity of chytrids. A substantial portion of chytrid diversity may be linked to algal parasitism, which is particularly noteworthy given the low algal diversity yet high algal biomass in these ecosystems. We detected 63 to 81 novel lineages distinct from previously described taxa (using the 97 or 98% identity threshold, respectively), assigned to the Blastocladiomycota and Monoblepharidomycota phyla, and four orders within the Chytridiomycota phylum (Rhizophydiales, Chytridiales, Lobulomycetales and Mesochytriales). Generally, most of the taxa were retrieved from dark ice and snow habitats (84.3%), relative to cryoconite (15.7%) (Table 1). Only two taxa were assigned to known species (Table 2).

Phylogenetic analysis of a concatenated dataset (18S-5.8S-28S rRNA) revealed the presence of taxa sharing a high percentage of the 18S identity (> 97 or 98%) retrieved from different sampled locations (SE, SW, CW and NW Greenland) and habitats (dark ice, red and green snow, and cryoconite) (Fig. 3). The same taxa also shared a high identity with uncultured species from other cold locations and different sample types (e.g. periglacial soil (Nepal and USA), sea ice (Sweden), glacial ice (Svalbard) or lake freshwater (Japan and France)) (Table 2). These results suggest that certain taxa of chytrids occur at different locations on the Greenland Ice Sheet and therefore that they have a wide geographical distribution (23.8%, with 98% identity, were shared between different locations). Most of the taxa found on the Greenland Ice Sheet have never previously been reported (76.8%, with 98% identity) (Table 2; Fig. 3).

Several environmental sequences that were recovered from dark ice, red and green snow were assigned to the Mesochytriales family. These taxa may be associated with glacier ice and snow algal communities, but their ecological roles remain uncertain. Currently, the only described member of Mesochytriales is an obligate algal parasite (*Mesochytrium penetrans*^50^. In addition, a recent study isolated chytrids parasitizing snow algae in alpine snow ecosystem in Japan, identified in the Mesochytriales order^49^ (Figure S2). Some of these sequences shared a high identity (98–98.7%) with our environmental sequences (SW_B taxon6, SE-SW taxon8, CW taxon 28, SW_A taxon6), implying that our sequences belong to chytrids infecting snow algae. Chytrids can be defined as specialist parasites (i.e., infecting only one host species), generalist parasites (i.e., infecting more than one host species), and facultative parasites (i.e., when it shows both lifestyles, parasitic and saprotrophic). Our data are consistent with the assertion that the Mesochytriales found here infect both snow algae - and glacier ice algae species.

A novel subspecies of *Urceomyces sphaerocarpus* (formerly known as *Rhizophidium sphaerocarpum*) was morphologically identified as the chytrid infecting the glacier ice algae *Ancylonema nordenskioldii* on the Columbia glacier in Alaska^29^. However, our environmental 18S sequences assigned to the Rhizophydiales showed only moderate similarity (78.7–93.3%) to the database sequences of such subspecies. One plausible explanation is that the chytrid species described by Kol^29^, based solely on morphological criteria, is not conspecific with later molecularly characterized *U. sphaerocarpus* isolates, which are currently described as saprotrophic strains. This highlights the well-recognized limitation of morphology-based chytrid taxonomy, as homoplasy in thallus morphology can obscure phylogenetic relationships and complicate species identification^51^. Consequently, linking historical morphospecies names and their inferred ecological roles to modern molecular lineages is inherently challenging and prone to misassignment, particularly in poorly sampled environments such as glacial ecosystems^52^. In addition, genetic variation in algal hosts can influence the susceptibility to infection^27,53^, and so it is likely that differences in parasite distribution could also reflect the genetic diversity of the host.

We found different numbers of potentially novel chytrids lineages distinguished from previously described taxa, depending on the similarity cut-off chosen. Generally, species definition and sequence similarity thresholds are difficult to define in any kingdom of life. This is particularly true for fungi, where there is no consensus on what the cut-off should be when it comes to fungal 18S sequences. The limitation of having a broader threshold for species definition is that known species in the Lobulomycetales family, *Clydaea vesicula* and *Lobulomyces angularis*, clustered as a single species (having an identity of 97.9% in the 18S gene sequence). A multi-loci approach and pure cultures are ideal when evaluating potentially novel species. Thus, our sequencing approach on 18S similarity allows us only a basic overview of potentially novel taxa in glacial habitats.

### Seasonal quantification and dynamics of active chytrids on dark ice and cryoconite habitats

The temporal dynamics over a 21-day period during the 2022 melt season in CW Greenland, showed that dark ice samples had a large variability in chytrid abundance (around one order of magnitude), while chytrid abundance in cryoconite samples from adjacent location was more stable (Fig. 5b), both temporally and spatially. The dynamic nature of the dark ice during a melt season, due to a range of weather events that alter both the physical properties and well as the hydrology of the dark ice, makes it challenging to pinpoint consistent seasonal patterns. The abundance and diversity of Chytridiomycota in habitats dominated by glacier ice algae can be attributed to the unique conditions created by algal blooms. High algal density and nutrient availability could benefit both chytrid lifestyles (parasitic and saprotrophic), while the thin layer of liquid water within the weathering crust, provides an ideal environment for zoospores to swim and infect various hosts.

The analysis of TotalRNA data enabled us to track the seasonal activity of the active chytrid community. The different active phyla and orders had distinct relative abundances (lower in cryoconite samples) with relatively similar temporal and spatial fluctuations (Fig. 4). In our 2022 samples from CW Greenland, Blastocladiomycota species were present only in the cryoconite samples, while they were present in dark ice and the pigmented snow collected in 2023 from NW Greenland. Spatial variability for Blastocladiomycota on the Greenland Ice Sheet appears thus to be greater compared to the Chytridiomycota. Members of the Blastocladiomycota phylum are both saprotrophic on decaying plants and animals, and either obligate or facultative parasites of invertebrates, plants, algae, oomycetes, and other blastoclads^54^. Blastoclads are globally distributed in numerous aquatic and terrestrial habitats; in cold regions, they are commonly found in polar and alpine lakes^55,56^, in glacial forefields, cryoconite hole water and sediment, supraglacial sediment, soil, ice and supraglacial water^57^.

Taxa within the Mesochytriales exhibited the highest relative abundances, and thus the greatest activity among chytrid orders, in dark ice samples, averaging between 1.28% and 1.56%. Their relative abundance was also notably higher than in cryoconite, where they averaged only 0.05% to 0.09%. These findings, supported by the phylogenetic analysis discussed in the previous subsection, suggest that chytrids infecting *Ancylonema *might belong to this order. Kobayashi et al.^31^, reported substantially higher infection rates of *Ancylonema* in cryoconite holes (20%) compared to surface ice (4%), suggesting that active parasitism may be more pronounced in cryoconite habitats. One possible interpretation is that the elevated relative abundance of *Mesochytriales* in dark ice reflects the presence of free-living or dispersal stages (e.g. zoospores) rather than active infections. Alternatively, differences between dark ice and cryoconite may reflect habitat-specific dynamics, including host availability, hydrological connectivity, or temporal variation in algal susceptibility, potentially influenced by environmental factors including weather conditions. Overall, these results emphasize that relative abundance alone does not directly indicate parasitic activity and highlight the need for combined molecular and microscopic approaches to accurately resolve habitat-specific host–chytrid interactions in supraglacial ecosystems. Given that Rhizophydiales, Lobulomycetales and Chytridiales include algal infecting species other than saprotrophic ones (^2,7,58–60^; Seto & Degawa^61^^62^,), we speculate that strains within these orders could potentially infect the glacier ice algae *Ancylonema*.

### Final remarks for future research

Our work highlights the presence of a remarkable diversity of previously undescribed, potentially new taxa of chytrids and their abundance in surface ice environments of the Greenland Ice Sheet. However, the challenges in accurately identifying and characterizing these chytrids limit our understanding of their interactions with their hosts and potential parasitic roles. Single cell genomic data of chytrid cells infecting *Ancylonema* sp. will clarify which species do infect the glacier ice algae and would possibly allow the design of species-specific primers, as done for chytrids infecting phytoplankton^63^. This can facilitate attempts to assess the temporal fluctuations of both host and parasite throughout the active season and clarify the role of chytrids as top-down controls in glacier ice algal blooms. Furthermore, future cultivation efforts will enable the study of the morphological and molecular characteristics of chytrid life stages, as well as providing insights into their host range and specificity with experimental cross-infection assays. Cultured isolates and/or targeted amplicon sequencing would further allow the generation of reliable ITS datasets, offering superior resolution for species-level delineation of chytrid diversity on the GrIS.

## Supplementary Information

Below is the link to the electronic supplementary material.

Supplementary Material 1

Supplementary Material 2

## Data Availability

The 2019–2020 dataset can be accessed through NCBI under PRJNA1011216. The 2021 dataset can be accessed through NCBI under PRJNA1011216. The 2022 dataset can be accessed through NCBI under PRJNA1160058. The relevant 2023 datasets as well as additional sequences, alignment, and tree files are available and can be accessed through Zenodo (v1.1) with the DOI [https://doi.org/10.5281/zenodo.16810413](https:/doi.org/10.5281/zenodo.16810413). Supplementary information, qPCR data and tables are available and can be accessed through Zenodo (v1.1) with the DOI [https://doi.org/10.5281/zenodo.16810413](https:/doi.org/10.5281/zenodo.16810413).
