# Supplementary material for: The diversity and abundance of chytrids on the Greenland Ice Sheet
Source: Sci Rep. 2026 Feb 26;16:11175. doi: 10.1038/s41598-026-41468-5 (PMC13046730; doi:10.1038/s41598-026-41468-5)
Supplement: Supplementary file 1 — Supplementary Material 1 [file 41598_2026_41468_MOESM1_ESM.docx]

The diversity and abundance of chytrids on the Greenland Ice Sheet – new quantitative data.

Authors: Laura Perini^1^, Athanasios Zervas^1*^, Louise Feld^1^, Carsten S. Jacobsen^1^, Liane G. Benning^2,3^, Martyn Tranter^1^, Alexandre M. Anesio^1^

^1^ Department of Environmental Science, Aarhus University, 4000 Roskilde, Denmark

^2^ GFZ, Helmholtz Centre for Geosciences, Telegrafenberg, 14473 Potsdam, Germany

^3^ Department of Earth Sciences, Freie Universität Berlin, 12249 Berlin, Germany

*Corresponding author: Athanasios Zervas ([az@envs.au.dk](mailto:az@envs.au.dk))


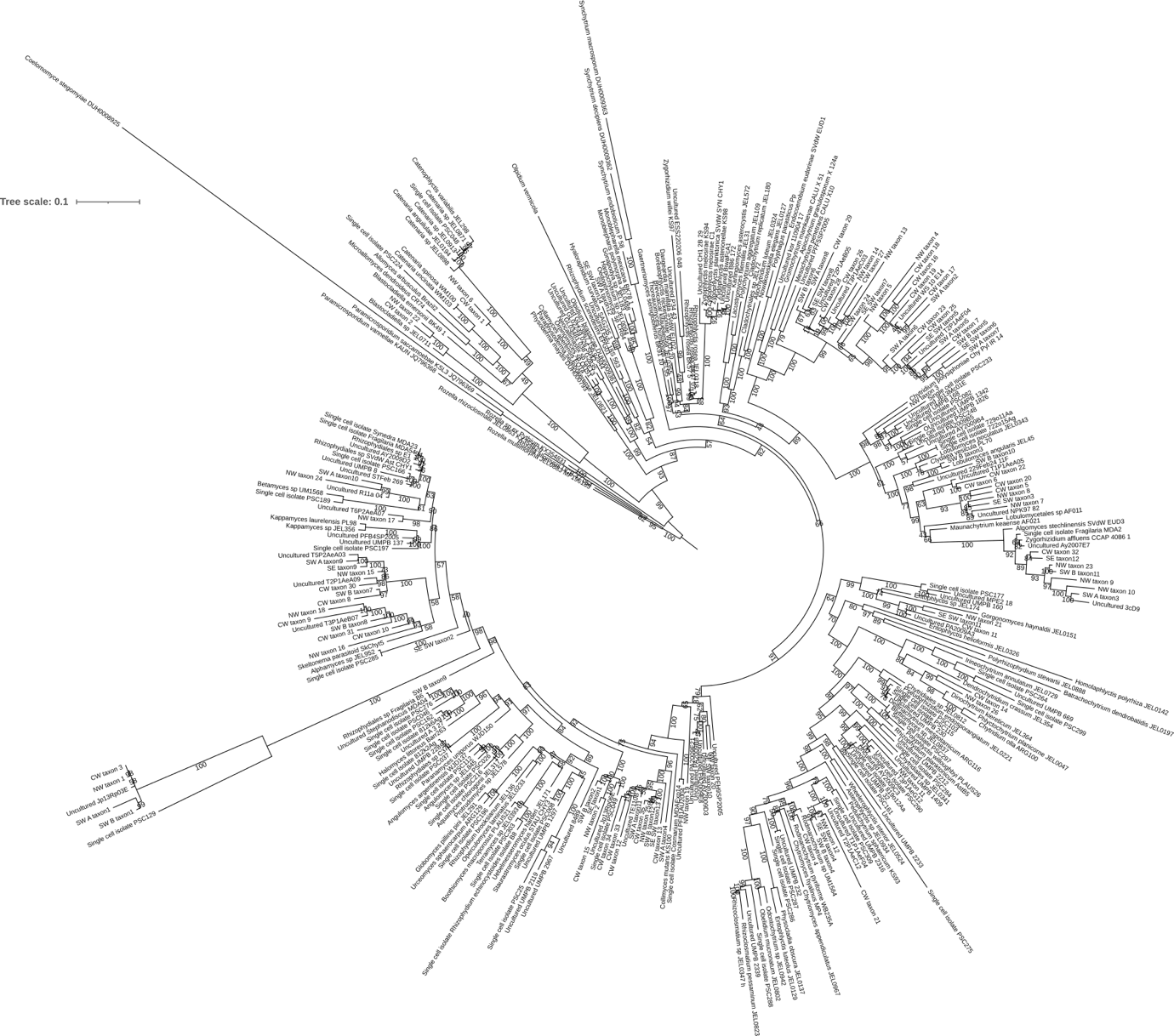


FigureS1 Maximum likelihood tree showing the phylogenetic position of chytrid species belonging to Blastocladiomycota, Monoblepharidomycota and Chytridiomycota phyla using the alignment of the 18S rRNA gene. The dataset of 18S rRNA sequences from 237 reference species and uncultured taxa published in Seto et al., 2023 was included. ML bootstrap values higher than 50% were shown on each branch.


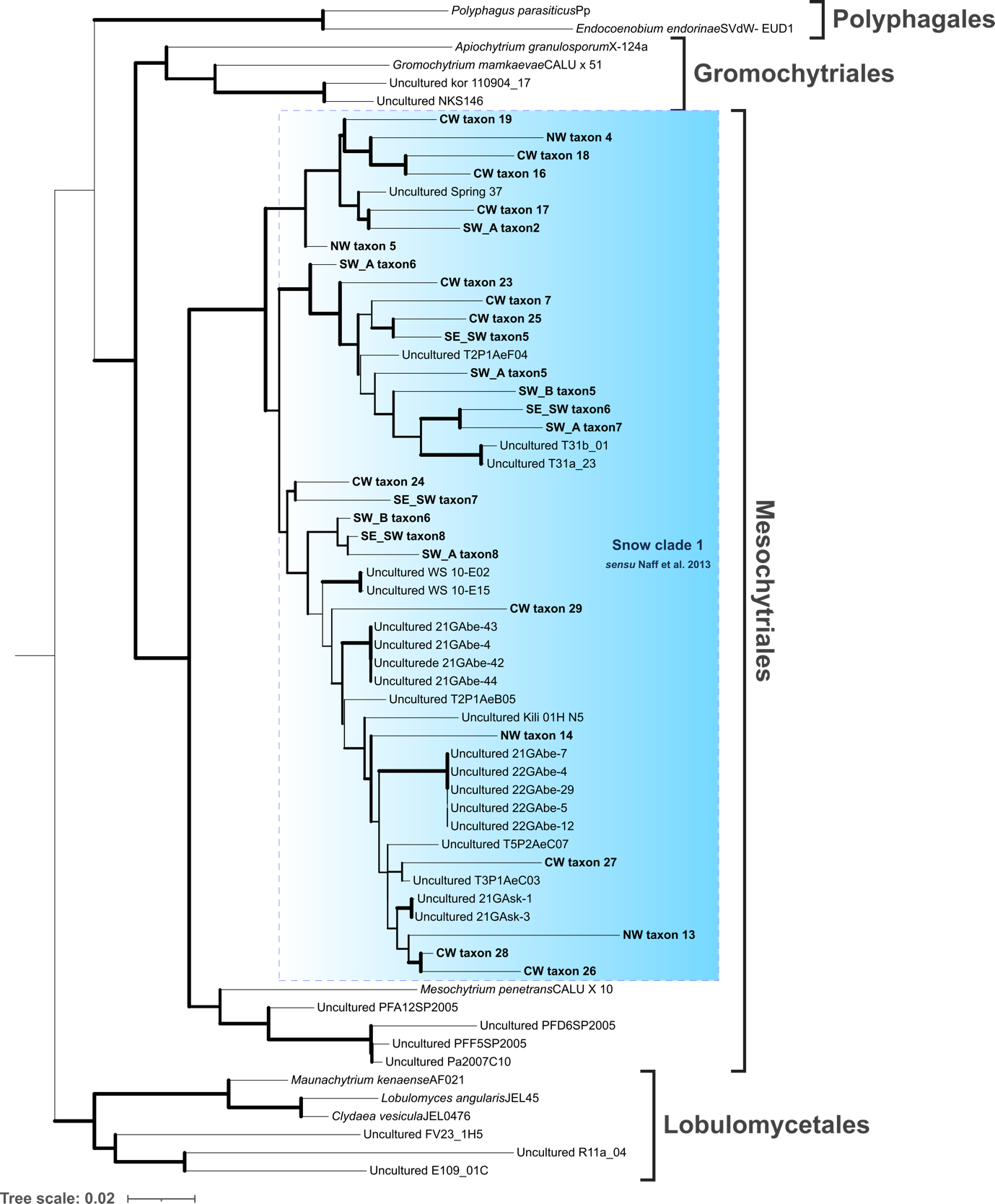


Figure S2 Maximum likelihood molecular phylogenetic tree showing the phylogenetic position of chytrid species belonging to Polyphagales, Gromochytriales, Mesochytriales, and Lobulomycetales using 18S rRNA. Lobulomycetales is the outgroup. Bold letters indicate samples detected in this study. Light blue shading indicates Snow Clade 1 proposed by Naff et al. (2013). Nodes supported by bootstrap ≥70% are highlighted with a bold line.
